# Patient Perceptions and Knowledge of Ionizing Radiation From Medical Imaging

**DOI:** 10.1001/jamanetworkopen.2021.28561

**Published:** 2021-10-13

**Authors:** Luca Bastiani, Fabio Paolicchi, Lorenzo Faggioni, Massimo Martinelli, Roberta Gerasia, Chiara Martini, Patrizia Cornacchione, Matteo Ceccarelli, Dante Chiappino, Daniele Della Latta, Jacopo Negri, Donatella Pertoldi, Donato Negro, Giovanni Nuzzi, Vincenzo Rizzo, Paola Tamburrino, Chiara Pozzessere, Giacomo Aringhieri, Davide Caramella

**Affiliations:** 1 Institute of Clinical Physiology of the Italian National Research Council, Pisa, Italy; 2 Diagnostic and Interventional Radiology, University of Pisa, Pisa, Italy; 3 Italian National Research Council Institute of Information Science and Technologies, Signals & Images Laboratory, Pisa, Italy; 4 Radiology Unit, Mediterranean Institute for Transplantation and Advanced Specialized Therapies, Palermo, Italy; 5 Department of Medicine and Surgery, University Hospital of Parma, Parma, Italy; 6 UOC Oncological Radiotherapy, Department of Diagnostic Imaging, Oncological Radiotherapy and Hematology, A. Gemelli University Hospital Foundation, Mediterranean Institute for Transplantation and Advanced Specialized Therapies, Rome, Italy; 7 Department of Physics, University of Cagliari, Calgiari, Italy; 8 Clinical Physiology of the Italian National Research Council/Institute of Materials, Cittadella Universitaria di Monserrato, Monserrato, Italy; 9 Department of Radiology, Institute of Clinical Physiology of the Italian National Research Council /Tuscany Region “Gabriele Monasterio Foundation,” Massa, Italy; 10 Monasterio Foundation, Tuscany Region “Gabriele Monasterio Foundation, Massa, Italy; 11 Now with TeraRecon Inc, Durham, North Carolina; 12 Department of Radiology, Macerata Hospital, Macerata, Italy; 13 Cardiovascular Diagnosis and Endoluminal Interventions Unit, Rovigo General Hospital, Rovigo, Italy; 14 Department of Medicine-DIMED University Hospital of Padua, Padua, Italy; 15 SantAnna and San Sebastiano Hospital, Caserta, Italy; 16 Nuclear Medicine Unit, San Giuseppe Moscati Hospital, Avellino, Italy; 17 Foggia United Hospitals, University Hospital of Foggia, Foggia, Italy; 18 Radiology Unit, AUSL Toscana Centro San Giuseppe Hospital, Empoli, Italy

## Abstract

**Importance:**

Although imaging has become a standard tool of modern medicine, its widespread use has been paralleled by an increasing cumulative radiation dose to patients despite technological advancements and campaigns calling for better awareness and minimization of unnecessary exposures.

**Objective:**

To assess patients’ knowledge about medical radiation and related risks.

**Design, Setting, and Participants:**

A survey study of hospitals in Italy was conducted; all patients in waiting rooms for medical imaging procedures before undergoing imaging examinations at 16 teaching and nonteaching hospitals were approached to take the survey. The survey was performed from June 1, 2019, to May 31, 2020.

**Main Outcomes and Measures:**

Survey respondents’ basic knowledge of ionizing radiation levels and health risks, earlier imaging tests performed, and information and communication about radiation protection issues.

**Results:**

Among 3039 patients invited to participate, the response rate was 94.3% (n = 2866). Participants included 1531 women (53.4%); mean (SD) age was 44.9 (17.3) years. Of the 2866 participants, 1529 (53.3%) were aware of the existence of natural sources of ionizing radiation. Mammography (1101 [38.4%]) and magnetic resonance imaging (1231 [43.0%]) were categorized as radiation-based imaging modalities. More than half of the 2866 patients (1579 [55.1%]; *P* = .03) did not know that chest computed tomography delivers a larger dose of radiation than chest radiography, and only 1499 (52.3%) knew that radiation can be emitted after nuclear medicine examinations (*P* = .004). A total of 667 patients (23.3%) believed that radiation risks were unrelated to age, 1273 (44.4%) deemed their knowledge about radiation risks inadequate, and 2305 (80.4%) preferred to be informed about radiation risks by medical staff. A better knowledge of radiation issues was associated with receiving information from health care professionals (odds ratio [OR], 1.71; 95% CI, 1.43-2.03; *P* < .001) and having a higher educational level (intermediate vs low: OR, 1.48; 95% CI, 1.17-1.88; *P* < .001; high vs low: OR, 2.68; 95% CI, 2.09-3.43; *P* < .001).

**Conclusions and Relevance:**

The results of this survey suggest that patients undergoing medical imaging procedures have overall limited knowledge about medical radiation. Intervention to achieve better patient awareness of radiation risks related to medical exposures may be beneficial.

## Key Points

**Question:**

What are the patient perceptions and knowledge about ionizing radiation used for medical imaging?

**Findings:**

In this survey study among 2866 patients undergoing radiological examinations in Italian hospitals, a substantial proportion of respondents perceived their medical radiation knowledge as inadequate and had misconceptions about basic aspects of radiation protection. Better knowledge was associated with receiving such information from medical staff and having a higher educational level.

**Meaning:**

These findings suggest that interventions to improve patients’ knowledge about radiation protection risks would be beneficial, with communication from medical staff potentially playing a determinant role.

## Teaser

This survey study examines patients’ knowledge of radiation used in imaging procedures.

## Introduction

Owing to the development and widespread availability of cross-sectional imaging, in the last several decades, radiology has become pivotal in the diagnosis and management of many diseases. The use of medical imaging, including ionizing radiation-based modalities, continues to increase, raising concerns about patients’ radiation exposure,^1,2,3,4^ with reported cumulative effective doses exceeding 100 mSv for single procedures.^5,6,7,8^ Although accounting for only 17% of all medical examinations, multidetector computed tomography (CT) alone makes up approximately 50% of the total radiation burden for medical purposes, and a large multicenter trial by Rehani et al^9^ revealed that more than 1% of patients undergoing multiple CT examinations over 1 to 5 years received a cumulative effective dose above 100 mSv.

The European Council Directive 2013/59/Euratom has emphasized the need for “safety standards for protection against the dangers arising from exposure to ionizing radiation.”^10^ Technical advances aimed to optimize radiation dose use, and awareness campaigns for health care professionals (including general practitioners, clinicians, radiologists, nuclear medicine physicians, and radiographers) and patients are the main ways to minimize unnecessary radiation exposure. Several studies have reported a lack of knowledge about medical radiation and related risks among both health care professionals and patients.^11,12,13,14,15,16^ In particular, patients’ knowledge about medical radiation is limited, and the perception of radiation risks is variable depending on age and educational level.^17,18,19^

In Italy, this issue is of special interest owing to the European Council Directive 2013/59/Euratom having become effective starting on August 27, 2020.^11,20^ In this context, a nationwide survey might help to more thoroughly assess patients’ knowledge about medical radiation and its potential risks. Our purpose was to develop and validate a questionnaire aimed to assess such knowledge among Italian patients and identify any differences related to patient sex, age, educational level, information received, and radiological procedures performed.

## Methods

### Population and Data Collection

A multicenter, nationwide survey study with prospective data collection was performed between June 1, 2019, and May 31, 2020, with patients in waiting rooms for medical imaging examinations in 16 Italian academic and nonacademic hospitals. Radiography students were trained as interviewers to achieve more generalized respondent comprehension and avoid misunderstanding. A web platform containing an informative brochure, a user’s guide, and the online questionnaire was implemented for easier collection of the survey results. Inclusion criteria were provision of written and individually signed patient informed consent, patient ability and willingness to adhere to all study requirements, and age 18 years or older. Exclusion criteria were mental illness, physical inability to respond and/or no or limited legal capacity, and age younger than 18 years.

Our study was approved by the regional ethical committee for clinical trials (Comitato Etico di Area Vasta Nord Ovest), and all of the involved radiology departments agreed to participate in the study. All patients gave their written informed consent to take the survey and were assured about the anonymity of responses; participants did not receive compensation. The survey was anonymously completed only once by each volunteer.

### Questionnaire Development

This study followed the American Association for Public Opinion Research (AAPOR) reporting guideline for survey studies. The survey consisted of 23 items grouped into 3 sections (eAppendix in the Supplement). The first section of the survey contained questions on factors such as sex, age, marital status, and educational level. The second section contained questions aimed to explore the patient’s knowledge about ionizing radiation risks (Knowledge About Ionizing Radiation Questionnaire [KIRQ]) and was divided into 3 steps. The first step identified survey questions (items generation) based on a review of current biomedical and life sciences literature (eTable 1 in the Supplement), which resulted in creation of the first KIRQ version. In the second step, semantic structure and content of each item in terms of statement relevance, clarity, and appropriateness were assessed by 5 radiologists with more than 10 years of experience in radiation protection policies (first questionnaire version). After modification and optimization of the selected items, 10 questions were included in the second version and validated by an independent panel of radiology educators. The third step involved pretesting the questionnaire on 20 nonmedical volunteers to assess its comprehensibility (pilot test). A reliability test-retest was performed on 50 volunteers and reapplied 2 weeks later to ensure the stability of questionnaire scores over time.^21^ The third section of the survey contained questions aimed to explore expectations and communication gaps between health care professionals and survey respondents The full survey questions are reported in the eAppendix in the Supplement.

### Statistical Analysis

Sample characteristics from the 3 questionnaire sections are assessed using descriptive statistics, whereas categorical variables are expressed as percentages and continuous variables are reported as mean (SD). The Italian regions were grouped into 3 territorial subareas: north, center, and south/islands.^22^ The educational level of the survey respondents was classified as low (ie, unschooled, primary school, or middle school), intermediate (ie, technical college [3 years] or high school/diploma), or high (ie, bachelor [3 years], master, or PhD degree).

We performed exploratory factor analysis and confirmatory factor analysis via structural equation modeling to identify latent factors underlying the psychometric properties of the 10-item KIRQ.^23^ To quantify the reliability of the questionnaire, we calculated the Cronbach index (α) as a measure of internal consistency. Several goodness-of-fit criteria were used, including the standardized root mean square residual, root mean square error of approximation (cutoff ≤0.10), comparative fit index, and Tucker-Lewis index (cutoff >0.90).^24,25^

Respondents’ scores were recorded using a binary classification of the KIRQ score with a 75th-percentile threshold separating high knowledge (≥75th percentile, binary value 1) from low to moderate knowledge (<75th percentile, binary value 0). Binary logistic models were applied to evaluate the association between personal data, communication, and information aspects and respondents’ knowledge. Setting the binary KIRQ score as the dependent variable and personal data, communication, and information items as independent variables, we performed univariable logistic regression as a first step to identify predictive variables and subsequently performed a multivariable logistic regression to simultaneously test the combinations of variables selected by univariable multinomial logistic regression (adjusted for sex and age). Results are reported as odds ratios (ORs) and their 95% CIs.

Statistical analysis was performed using Stata/SE, version 15 (StataCorp LLC) and SPSS, version 24 (IBM Corp). All *P* values were 2-sided, and *P* < .05 was set as the threshold for statistical significance.

## Results

Our survey was conducted among 3039 individuals, with a response rate of 94.3% (n = 2866). The sample population included 1531 women (53.4%) and 1335 men (46.6%); mean (SD) age was 44.9 (17.3) years. The survey had a homogeneous geographic distribution (north, 1139 [39.7%], center, 889 [31.0%], and south/islands, 838 [29.2%]; *P* = .06).

The educational level of survey respondents was low in 661 respondents (23.1%), intermediate in 1367 (47.7%), and high in 838 (29.2%). Most respondents were married (1404 [49.0%]). Surveyed women had a higher level of education (491 [32.1%]) than men (347 [26.0%]) (Table 1).

**Table 1.** Personal Data of Survey Respondents

Variable	No. (%)			*P* value
	Men (n = 1335)	Women (n = 1531)	Total (n = 2866)	
Age, mean (SD), y	45.44 (17.4)	44.35 (17.2)	44.90 (17.3)	.10
Geographic area				
North	520 (29.7)	619 (32.2)	1139 (39.7)	.06
Center	396 (39.0)	493 (40.4)	889 (31.0)	
South/islands	419 (31.4)	419 (27.4)	838 (29.2)	
Nationality				
Italian	1283 (96.1)	1466 (95.8)	2749 (95.9)	.71
Other	52 (3.9)	65 (4.2)	117 (4.1)	
Educational level				
Low	330 (24.7)	331 (21.6)	661 (23.1)	<.001
Intermediate	658 (49.3)	709 (46.3)	1367 (47.7)	
High	347 (26.0)	491 (32.1)	838 (29.2)	
Marital status				
Unmarried/civil partnership	506 (37.9)	580 (37.9)	1086 (37.9)	.46
Married	665 (49.8)	739 (48.3)	1404 (49.0)	
Separated/divorced	116 (8.7)	117 (7.6)	233 (8.1)	
Widowed	48 (3.6)	95 (6.2)	143 (5.0)	

**Figure 1.**
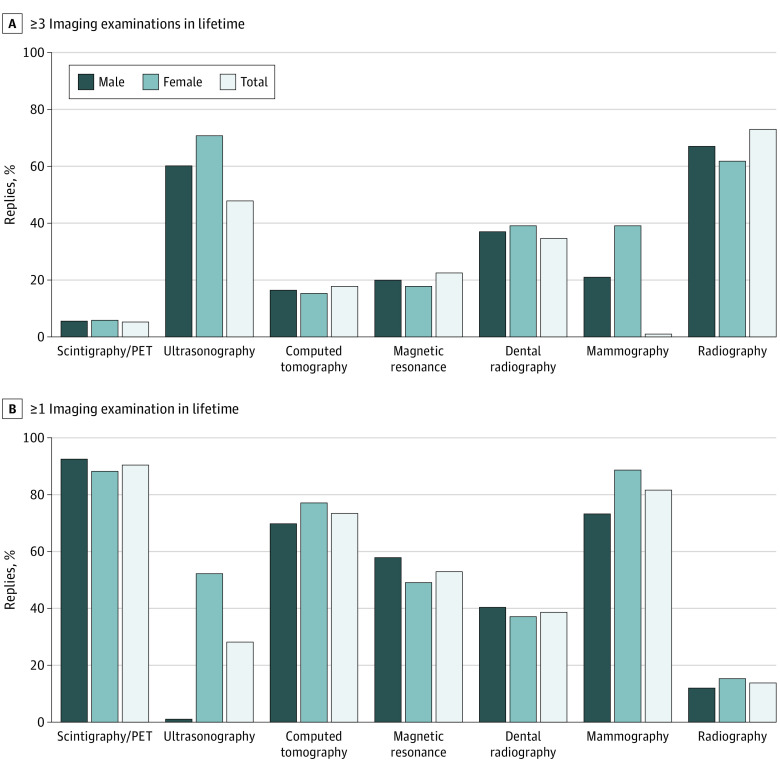
Lifetime Imaging Tests of Survey Respondents Responses of individuals who had received 3 or more imaging examinations (A) and 1 or more imaging examinations (B). PET indicates positron emission tomography.

Almost all 2866 survey respondents (2823 [98.5%]) reported having undergone at least 1 radiological test in their lifetime. Of the 42 respondents (1.5%) undergoing an imaging examination for the first time, 1.3% were women (20/1531) and 1.6% were men (22/1335) (mean [SD] age, 32.1 [14.5 years]). Most of them had undergone examinations involving ionizing radiation at least once (Figure 1), including radiography (1244 men [93.2%], 1358 women [88.7%], 2602 combined [90.8%]), dental radiography (936 men [70.1%], 1185 women [77.4%], 2021 combined [74%]), CT (545 men [40.8%], 574 women [37.5%], 1119 combined [39%]), mammography (17 men [1.3%], 804 women [52.5%], 821 combined [28.6%]), and nuclear medicine imaging (164 men [12.3%], 237 women [15.5%], 401 combined [14%]). At least 3 exposures to radiography examinations had been performed in 1916 (66.8%) respondents (973 men [72.9%], 943 women [61.6%]); dental radiography, 1058 respondents (36.9%) (462 men [34.6%], 596 women [38.9%]); CT, 468 respondents (16.3%) (235 men [17.6%], 233 women [15.2%]); mammography, 601 respondents (21%) (11 men [0.8%], 594 women [38.8%]); and nuclear medicine, 158 respondents (5.5%) (69 men [5.2%], 89 women [5.8%]). Among radiation-free imaging tests, ultrasonography had been performed at least once in 2345 respondents (81.9%) (984 men [73.7%], 1361 women [88.9%]), whereas magnetic resonance imaging (MRI) had been performed at least once in 1532 respondents (53.4%) (776 men [58.1%]; 756 women [49.4%]). The mean (SD) overall number of lifetime imaging tests was slightly higher in women (4.31 [1.8]; median, 4; IQR, 3-6) than men (3.8 [1.7]; median, 4; IQR, 3-5) (*P*<.001).

In the KIRQ survey section, 1529 of the 2866 respondents (53.3%) were aware of the existence of natural sources of ionizing radiation, with no statistically significant difference between men and women (Table 2). Computed tomography was correctly categorized as radiation-based imaging by 2035 respondents (71.0%), and mammography was correctly categorized by 1101 respondents (38.4%). Although no statistically significant difference was found in the rate of correct answers about CT between men and women, correct answers regarding mammography were provided by women (711 of 1531 [46.4%]) more frequently than men (390 of 1335 [29.2%]) (*P* < .001).

**Table 2.** Respondents’ Replies to the Second and Third Survey Sections^a^

Survey question	No. (%)			P value
	Men (n = 1335)	Women (n = 1531)	Total (N = 2866)	
**Category B: Knowledge**				
B4. Is there a natural source of ionizing radiation to which we are all exposed?				
Yes/no	721 (54.0)	808 (52.8)	1529 (53.3)	.51
B5. Which of these radiological examinations involve exposure to ionizing radiation? (multiple choices allowed)				
Ultrasonography	1096 (82.1)	1340 (87.5)	2436 (85.0)	<.001
CT^b^	964 (72.2)	1071 (70.0)	2035 (71.0)	.18
Magnetic resonance^b^	581 (43.5)	650 (42.5)	1231 (43.0)	.57
Mammography	390 (29.2)	711 (46.4)	1101 (38.4)	<.001
B6. Which of the following imaging tests delivers a higher radiation dose?				
Chest CT^b^	568 (42.5)	719 (47.0)	1287 (44.9)	<.05
Chest radiograph	395 (29.6)	393 (25.7)	788 (27.5)	
The amount of radiation is the same	372 (27.9)	419 (27.4)	791 (27.6)	
B7. Following which radiological tests can one emit radiation (even some time after it)?				
Contrast-enhanced ultrasonography	62 (4.6)	63 (4.1)	125 (4.4)	<.01
Contrast-enhanced CT	230 (17.2)	243 (15.9)	473 (16.5)	
Scintigraphy^b^	648 (48.5)	851 (55.6)	1499 (52.3)	
All of the above	226 (16.9)	208 (13.6)	434 (15.1)	
None of the above	169 (12.7	166 (10.8)	335 (11.7)	
B8. For an abdominal CT scan, how does the amount of radiation dose delivered to a thinner patient (60 kg) compare with that delivered to a heavier one (100 kg)?				
Higher in a thin patient	186 (13.9)	225 (14.7)	411 (14.3)	.24
Higher in a heavy patient^b^	444 (33.3)	464 (30.3)	908 (31.7)	
It is comparable	705 (52.8)	842 (55.0)	1547 (54.0)	
B9. How dangerous is it to undergo radiological tests using ionizing radiation?				
Not very dangerous^b^	552 (41.3)	471 (30.8)	1023 (35.7)	<.001
Quite dangerous	639 (47.9)	865 (56.5)	1504 (52.5)	
Very dangerous	144 (10.8)	195 (12.7)	339 (11.8)	
B10. For which of the following is it riskier to undergo a radiological test using ionizing radiation?				
A child^b^	766 (57.4)	913 (59.6)	1679 (58.6)	<.01
A 25-y-old man	22 (1.6)	23 (1.5)	45 (1.6)	
A 25-y-old woman	90 (6.7)	97 (6.3)	187 (6.5)	
A middle-aged adult	38 (2.8)	34 (2.2)	72 (2.5)	
An older individual	128 (9.6)	88 (5.7)	216 (7.5)	
No difference (the risk is comparable)	291 (21.8)	376 (24.6)	667 (23.3)	
**Category C: Communication**				
C1. How do you evaluate your knowledge about the risks associated with the use of ionizing radiation for medical purposes?				
Fair/good/excellent	338 (25.3)	377 (24.6)	715 (24.9)	.52
Sufficient	419 (31.4)	459 (30.0)	878 (30.6)	
Inadequate	578 (43.3)	695 (45.4)	1273 (44.4)	
C2. From which communication channels have you usually received information about the risks associated with the use of ionizing radiation for medical purposes?				
Television/radio	395 (29.6)	395 (25.8)	790 (27.6)	.02
Magazines/newspapers	201 (15.1)	245 (16.0)	446 (15.6)	.49
Internet or social media (eg, Facebook)	344 (25.8)	382 (25.0)	726 (25.3)	.62
Booklets	194 (14.5)	283 (18.5)	477 (16.6)	<.01
School, university	217 (16.3)	319 (20.8)	536 (18.7)	<.01
I have never received any information about ionizing radiation	488 (36.6)	514 (33.6)	1002 (35.0)	.10
C3. If you underwent a diagnostic examination with ionizing radiation, did you receive information about the risks associated with the use of ionizing radiation for that examination?				
Yes	551 (41.3)	671 (43.8)	1222 (42.6)	.21
I have never received any information about ionizing radiation	784 (58.7)	860 (56.2)	1644 (57.4)	
C4. From which of the following sources would you like to receive information regarding the risks associated with the use of ionizing radiation for medical purposes?				
Television/radio	581 (43.5)	597 (39.0)	1178 (41.1)	.01
Newspapers	325 (24.3)	346 (22.6)	671 (23.4)	.27
Internet or social media (eg, Facebook)	386 (28.9)	438 (28.6)	824 (28.8)	.86
Booklets	351 (26.3)	464 (30.3)	815 (28.4)	.02
School	407 (30.5)	513 (33.5)	920 (32.1)	.08
Health care staff	1057 (79.2)	1248 (81.5)	2305 (80.4)	.12
C5. In the health care environment, from which professional would you prefer to receive information about the risks associated with the use of ionizing radiation?				
Radiologist	905 (67.8)	1061 (69.3)	1966 (68.6)	.39
Medical physicist	183 (13.7)	177 (11.6)	360 (12.6)	.08
Radiographer	675 (50.6)	830 (54.2)	1505 (52.5)	.06
General practitioner	759 (56.9)	854 (55.8)	1613 (56.3)	.56
C6. At the end of a radiological examination, in which terms would you prefer to be informed about the amount of radiation received?				
The radiation value expressed in terms of radiation units	513 (38.4)	502 (32.7)	1015 (35.4)	.002
The equivalent risk to that of a No. of smoked cigarettes	515 (38.6)	448 (29.3)	963 (33.6)	<.001
The equivalent risk to that of a No. of days of background radiation exposure	427 (32.0)	555 (36.3)	982 (34.3)	.02
The equivalent risk to a No. of kilometers traveled by car	193 (14.5)	250 (16.3)	443 (15.5)	.17
I don’t want to be informed	142 (10.6)	199 (13.0)	341 (11.9)	.05

Abbreviations: CT, computed tomography; KIRQ, Knowledge About Ionizing Radiation Questionnaire.

^a^ Second section, KIRQ; third section, communication and information.

^b^ Correct answer for the KIRQ questions.

Ultrasonography was correctly classified as a radiation-free examination by 2436 respondents (85.0%), and MRI was correctly classified by 1231 respondents (43.0%). Women (1340 of 1531 [87.5%]) replied correctly to questions about ultrasonography more frequently than men (1096 of 1335 [82.1%]) (*P* < .001), whereas no statistically significant difference between men and women was found related to questions about MRI.

More than half of the 2866 respondents (1579 [55.1%]; *P* = .03) did not know that chest CT delivers more radiation compared with chest radiography, and slightly more than half knew that patients can emit radiation after nuclear medicine examinations (1499 of 2866 [52.3%]; *P* = .004). The question about the association between CT dose and patient’s body weight was correctly answered by only 31.7% (908) of the respondents, whereas 58.6% (1679) of respondents correctly replied to the question about radiation risks and age. Few respondents (667 [23.3%]) believed that radiation exposure risks were not affected by age.

In the third survey section, 1273 of the 2866 respondents (44.4%) deemed their knowledge about radiation risks as inadequate. They had been informed about radiation risks by radio and television (790 [27.6%]) exposure and from the internet, including Facebook or other social media (726 [25.3%]).

Approximately one-third (1002 [35.0%]) of the respondents had never been informed about ionizing radiation from mass media. However, most respondents (2305 [80.4%]) expressed the preference to receive such information from health care professionals.

Only 1224 respondents (42.7%) had been informed about radiation risks during an imaging examination. Most patients would like to be informed by a radiologist (1966 [68.6%]), followed by their general practitioner (1613 [56.3%]), a radiographer (1505 [52.5%]), and a medical physicist (360 [12.6%]).

A total of 2525 of the 2866 respondents (88.1%) wanted to be informed about the amount of radiation received after the radiological examination was completed. The most appreciated methods of communication were via a quantitative dose measurement using a specific radiation unit (eg, millisieverts) (1015 [35.4%]) and by expressing the radiation hazard in terms of equivalent cancer risk from a given number of smoked cigarettes (963 [33.6%]) (Table 2).

### Psychometric Properties of the 10-Item KIRQ

Exploratory factor analysis highlighted the presence of 1 latent factor underlying the 10-item questionnaire on ionizing radiation knowledge. The questionnaire showed acceptable internal consistency (Cronbach α, 0.742) (eTable 2 in the Supplement).

Confirmatory factor analysis revealed that the items related to ionizing radiation exposure from specific imaging modalities (question B5) were the most important variables, with a value equal to 0.478 for CT and 0.382 for MRI. Other variables with optimal specific validity index values were those related to the amount of radiation in association with specific imaging examinations (question B6; 0.374), natural sources of ionizing radiation (question B4; 0.342), and radiation emission after radiological tests (question B7; 0.313).

### Factors Associated With Knowledge of Radiation Protection

The KIRQ score greater than or equal to the 75th percentile was equal or above 7 points and the median was 5 points. The KIRQ score was significantly lower in patients older than 65 years (median, 4; IQR, 3-6 years) than in those aged 18 to 64 years (median, 5; IQR, 4-7) (*P* < .001).

Univariable analysis (Table 3) showed a significant positive association between ionizing radiation knowledge (KIRQ score ≥75th percentile) and age (OR, 0.99; 95% CI, 0.989-0.998; *P* = .01). A higher level of knowledge was associated with higher levels of education (intermediate vs low: OR, 1.48; 95% CI, 1.168-1.881; *P* < .001; high vs low: OR, 2.68; 95% CI, 2.091-3.426; *P* < .001). Respondents with high levels of self-perceived knowledge had greater knowledge about ionizing radiation (good or excellent vs inadequate self-perception knowledge: OR, 3.46; 95% CI, 2.789-4.279; *P* < .001). Respondents who had received information from health care professionals (OR, 1.71; 95% CI, 1.431-2.034; *P* < .001) or those who had sought information from brochures, radio, internet, or other mass media (OR, 1.36; 95% CI, 1.212-1.518; *P* < .001) were more knowledgeable about ionizing radiation. No significant association was found between ionizing radiation knowledge and respondents’ sex (OR, 0.91; 95% CI, 0.771-1.079; *P* = .28), lifetime number of radiological examinations (OR, 1.72; 95% CI, 0.762-3.898; *P* = .19), and geographic location (center vs south/islands: OR, 1.14; 95% CI, 0.923-1.407; *P* = .22; north vs south/islands: OR, 0.77; 95% CI, 0.623-1.045; *P* = .06).

**Table 3.** Univariable and Multivariable Logistic Regression

Variable	Univariable		Multivariable	
	OR (95% CI)	*P* value	OR (95% CI)	*P* value
Sex	0.91 (0.771-1.079)	.28	0.86 (0.718-1.035)	.11
Age	0.99 (0.989-0.998)	<.01	0.99 (0.987-0.999)	.02
Educational level				
Low	1 [Reference]		1 [Reference]	
Intermediate	1.48 (1.168-1.881)	<.001	1.42 (1.085-1.846)	.01
High	2.68 (2.091-3.426)	<.001	2.55 (1.933-3.359)	<.001
Self-perceived knowledge				
Inadequate	1 [Reference]		1 [Reference]	
Sufficient	2.30 (1.863-2.837)	<.001	2.01 (1.602-2.516)	<.001
Good or excellent	3.46 (2.789-4.279)	<.001	2.93 (2.315-3.708)	<.001
KIRQ information^a^				
B1. No. of imaging examinations (at least 1 in lifetime)	1.72 (0.762-3.898)	.19	1.51 (1.110-2.061)	.009
C3. Information received from health care professionals	1.71 (1.431-2.034)	<.001	1.30 (1.072-1.565)	.007
B2. No. of imaging examinations (≥3 in lifetime)	1.30 (1.002-1.691)	<.05	NA	NA
C2. Information about ionizing radiation received	1.36 (1.212-1.518)	<.001	NA	NA
C4. Information about ionizing radiation desired	1.07 (1.015-1.133)	.01	NA	NA
Geographic area			NA	NA
South/islands	1 [Reference]		NA	NA
Center	1.14 (0.923-1.407)	.22	NA	NA
North	0.77 (0.623-1.045)	.06	NA	NA

Abbreviations: KIRQ, Knowledge About Ionizing Radiation Questionnaire; NA, not applicable; OR, odds ratio.

^a^ The survey consisted of 23 items grouped into 3 sections. Items labeled as “B” indicate knowledge, and items labeled as “C” indicate communication.

**Figure 2.**
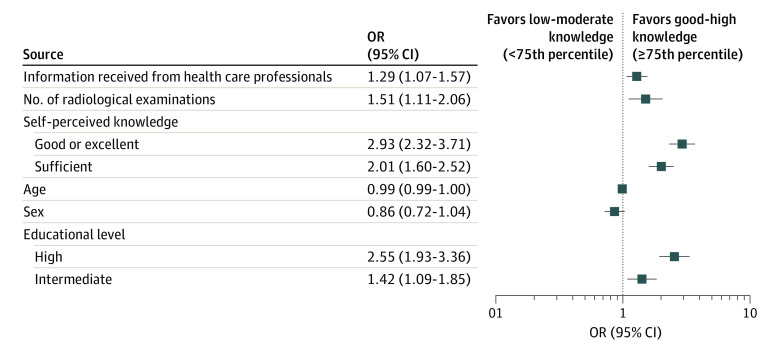
Multivariable Logistic Regression Analysis The reference category for educational level was low level, and the reference category for self-perceived knowledge was inadequate knowledge. OR indicates odds ratio.

Multivariable logistic regression (Figure 2) showed that higher educational level (intermediate vs low: OR, 1.42; 95% CI, 1.085-1.846; *P* < .01; high vs low: OR, 2.55; 95% CI, 1.933-3.359; *P* < .001) and sufficient or good self-perception of knowledge (sufficient vs inadequate: OR, 2.01; 95% CI, 1.602-2.516; *P* < .001; good or excellent vs inadequate: OR, 2.93; 95% CI, 2.315-3.708; *P* < .001) were associated with greater knowledge of ionizing radiation issues (KIRQ score ≥75th percentile). Statistically significant associations were also observed for lifetime number of radiological examinations (OR, 1.51; 95% CI, 1.110-2.061; *P* < .01) and information received from health care professionals (OR, 1.30; 95% CI, 1.072-1.565; *P* < .01). No statistically significant association was observed for sex, whereas younger age was associated with a greater knowledge level (OR, 0.99; 95% CI, 0.987-0.999; *P* = .02).

Overall, multivariable analysis showed that the most relevant factors associated with ionizing radiation knowledge were a higher educational level, an adequate self-perception of radiation knowledge, a higher number of imaging examinations performed, and having received radiation information from a health care professional. Although exposure to radiation information from various news sources (eg, television, radio, and newspapers) was also associated with a higher radiation knowledge on univariate analysis, such association was not confirmed by multivariate analysis.

## Discussion

Despite the fact that 98.5% of the respondents had undergone imaging, few had an understanding of radiation dose or risk and had gained their knowledge from outside the health care system, and most requested more information. These results suggest the need for new strategies even with the presence of many public radiation protection and awareness campaigns for more than a decade.

This study was designed to be a national-level survey of adults undergoing medical imaging procedures aimed to evaluate their knowledge and awareness about ionizing radiation, with the dual purpose of understanding their concerns and informing policy makers and radiation protection professionals about gaps in education and training of all stakeholders. To our knowledge, this is the first multicenter survey on this topic performed on a large population, as studies published so far have included a limited number of patients and/or single imaging centers.^17,18,26^

Our results revealed a lack of knowledge in the general population about radiation doses associated with common radiological examinations and basic radiation protection issues. In particular, the existence of natural background radiation and the dose burden of the most frequent imaging examinations were largely unknown by our surveyed patients. This lack of information was not due to having no experience with radiological procedures. In fact, nearly all of the respondents had previously received at least 1 imaging examination, including radiation-free imaging modalities, and about two-thirds of the patients had undergone at least 3 imaging tests; there was a prevalence of men in the number of total radiological examinations (72.9%), whereas women had undergone ultrasonographic examinations more frequently than other imaging tests, including mammography. Such a female prevalence of ultrasonographic examinations could be due to breast screening and follow-up testing for urogynecological concerns.

Although most patients had undergone an ultrasonographic or MRI examination at least once, most were unaware of the absence of ionizing radiation in MRI (57%), and to a lesser extent, this finding also applied to ultrasonography (15%). Moreover, 71.0% of surveyed patients knew that CT relies on ionizing radiation, whereas only 38.4% (men and women combined) knew that mammography uses ionizing radiation, with women knowing that mammography is radiation based more frequently than men (46.4% vs 29.2%; *P* < .001). To this latter point, a questionnaire-based study conducted by Hollada et al^27^ on 1725 patients presenting for a mammogram showed that, although 65% of the patients responded that they had been informed of the risks and benefits of the examination, 60% overestimated the amount of radiation in a mammogram, suggesting that targeted patient education for those undergoing any type of imaging procedure should be heightened. More generally, our findings are in line with those from previous studies, revealing an unmet need for awareness campaigns about medical radiation addressed to the general population.^26,27,28,29,30^ Efforts to improve patient awareness about CT and radiation protection have yielded some results,^28^ yet much remains to be done. In addition, our findings highlighted patients’ limited knowledge about the association between body mass index and delivered CT radiation dose, with more than half believing that radiation dose is unrelated to body size, and about the association between patient age and radiation risk.

Furthermore, most surveyed patients were unaware of the potential radiation risks to which they may have been exposed if their imaging had required ionizing radiation, with more than half of the respondents receiving no radiation information before, during, or after imaging examinations despite more stringent legal requirements about delivering patient information and recording and reporting of doses on medical procedures. Improved communication between medical staff and patients may be useful if it is the main and mandatory focus of further informative campaigns because more than 40% of the respondents had received information about ionizing radiation mainly from mass media and deemed the knowledge received from those sources to be inadequate. Approximately 80% of the respondents stated they would like to receive information from medical staff, attesting to their willingness to be properly informed about the potential risks of radiological procedures.

In particular, most patients (68.6%) would like to be given information on this topic by radiologists. This preference could be explained by assuming that patients prefer to be informed by physicians who, owing to the medical and technological skills that pertain to their professional specialty, will supervise their imaging procedures and interpret the findings in combination with their clinical history to reach a diagnosis, hence being directly involved in clinical management and communication with other medical specialists. Although to a lesser extent, patients would also like to be informed by their general practitioners, with whom they usually have a closer relationship than with other health care professionals, or by radiographers, probably owing to their specific technical expertise. Nonetheless, several factors may align to prevent satisfactory communication between health care professionals and patients in a busy radiology setting.^26,31^ It could be hypothesized that this communication gap may partly result from a heavy workload in radiological departments, often making it difficult for the radiological staff to provide patients with exhaustive information about radiation exposure. Several studies have highlighted patients’ preference to speak directly with imaging experts about their imaging findings,^19,32,33,34,35^ further emphasizing the need for improved direct communication between radiological staff and patients.

In line with Hartwig et al,^31^ who found an association between patients’ educational level and awareness of potential negative effects from medical imaging, our study found that educational level, self-perceived knowledge about ionizing radiation, and number of imaging examinations performed were associated with a higher degree of awareness and knowledge about the risks of radiation exposure. Moreover, the fact that these patients were more likely to seek radiation protection information through mass media might suggest that proper information given by the radiological staff at imaging appointments could trigger patients’ interest about radiation protection issues. This practice could possibly improve patient knowledge and awareness and, in the long run, ease the educational tasks of radiology personnel.

### Limitations

The study has limitations. The first limitation of our survey is that it was administered in waiting rooms of different hospitals without any differentiation based on imaging modality, potentially introducing a selection bias in the recruitment of survey respondents. Second, the sample selection and total number of respondents were not representative of the general patient population. Third, although our survey included a question on whether respondents who had undergone prior imaging examinations had been informed about radiation risks, it lacked more specific questions to assess whether such information was adequate. Fourth, although the survey had been tested and validated before being distributed to our final patient population, it cannot be considered as a standardized tool. Further studies with larger samples are needed, possibly via a nationwide or, even better, international standardized questionnaire, to get a more accurate representation of the general population’s radiation knowledge and awareness.

## Conclusions

The findings of our survey suggest a substantial lack of knowledge about medical radiation among Italian patients. This scenario calls for improved communication between medical staff and patients to provide them with adequate awareness about medical radiation and the risks related to cumulative radiation exposure.

## Notes

Supplement. **eAppendix.** Survey Questions **eTable 1.** Literature References Used for Generation of the Survey Items **eTable 2.** Psychometric Properties of the 10-Item KIRQ
