# Error in Affiliation

**DOI:** 10.1001/jamanetworkopen.2021.41299

**Published:** 2021-12-02

In the Original Investigation titled “Patient Perceptions and Knowledge of Ionizing Radiation From Medical Imaging,”^1^ published October 13, 2021, there was an error in an author affiliation. The affiliation for Patrizia Cornacchione, RT, included “Mediterranean Institute for Transplantation and Advanced Specialized Therapies,” but should have omitted that phrase to be given in its entirety as “UOC Oncological Radiotherapy, Department of Diagnostic Imaging, Oncological Radiotherapy and Hematology, A. Gemelli University Hospital Foundation, Rome, Italy.” This article has been corrected.^1^
