# Supplementary material for: Patient Perceptions and Knowledge of Ionizing Radiation From Medical Imaging
Source: JAMA Netw Open. 2021 Oct 13;4(10):e2128561. doi: 10.1001/jamanetworkopen.2021.28561 (PMC8515210; doi:10.1001/jamanetworkopen.2021.28561)
Supplement: Supplement. — eAppendix. Survey Questions eTable 1. Literature References Used for Generation of the Survey Items eTable 2. Psychometric Properties of the 10-Item KIRQ [file jamanetwopen-e2128561-s001.pdf]

Supplementary Online Content

Bastiani L, Paolicchi F, Faggioni L, et al. Patient perceptions and knowledge of ionizing radiation from medical imaging. *JAMA Netw Open*. 2021;4(10):e2128561. doi:10.1001/jamanetworkopen.2021.28561

eAppendix. Survey Questions

eTable 1. Literature References Used for Generation of the Survey Items

eTable 2. Psychometric Properties of the 10-Item KIRQ

This supplementary material has been provided by the authors to give readers additional information about their work.

© 2021 Bastiani L et al. *JAMA Network Open.*

eAppendix. Survey Questions

A. Personal data

A1. Gender

1. male
2. female

A2. Age _______

A3. Which language is mainly spoken in your family of origin?

1. Italian
2. Other

A4. Which is your marital status?

1. Unmarried
2. Civil partnership
3. Married
4. Separated
5. Divorced
6. Widowed

A5. Whom have you mostly lived in the last 12 months with?

1. Partner/cohabitee
2. Parents/relatives
3. Friends
4. Alone

A6. What is your qualification level?

1. None
2. Primary school
3. Secondary school

© 2021 Bastiani L et al. *JAMA Network Open.*

4. College
5. Bachelor's degree
6. Master’s degree
7. Postgraduate degree

A7. What is your employment?

1. Employed, permanent work
2. Employed, temporary work
3. Freelance
4. Unemployed
5. Housewife/househusband
6. Unable to work
7. Student
8. Retired

B. Knowledge

B1. Which of the following radiological tests have you undergone in your lifetime? (multiple choices allowed)

1. Radiography
2. Mammography
3. Dental X-ray
4. Magnetic resonance
5. Computed tomography
6. Ultrasound
7. Scintigraphy/PET

B2. Which of the following radiological tests have you undergone more than three times in your lifetime? (multiple choices allowed)

1. Radiography
2. Mammography
3. Dental X-ray
4. Magnetic resonance
5. Computed tomography
6. Ultrasound
7. Scintigraphy/PET

B3. Do you have any children under the age of 14 years old who have undergone radiological examinations?

1. Yes
2. No
3. I have no children

B4. In your opinion, is there a natural source of ionizing radiation which we are all exposed to?

1. **YES**
2. No

© 2021 Bastiani L et al. *JAMA Network Open.*

B5. Which of these radiological examinations involve exposure to ionizing radiation? (multiple choices allowed)

1. Ultrasound
2. **COMPUTED TOMOGRAPHY**
3. Magnetic resonance
4. **MAMMOGRAPHY**

B6. Which of the following imaging tests delivers a higher radiation dose?

1. **CHEST CT**
2. Chest X-ray
3. The amount of radiation is the same

B7. Following which radiological tests can one emit radiation (even some time after it)?

1. Contrast enhanced ultrasound
2. Contrast enhanced CT
3. **SCINTIGRAPHY**
4. All of the above
5. None of the above

B8. For an abdominal CT scan, how does the amount of radiation dose delivered to a thinner patient (60kg weight) compare to that delivered to a larger one (100kg weight)?

1. Higher in the thinner patient
2. **HIGHER IN THE LARGER PATIENT**
3. It is comparable

B9. How dangerous is it to undergo radiological tests using ionizing radiation?

1. **NOT VERY DANGEROUS**
2. Quite dangerous
3. Very dangerous

B10. For which of the following is it riskier to undergo a radiological test using ionizing radiation?

© 2021 Bastiani L et al. *JAMA Network Open.*

1. **A CHILD**
2. A 25-year-old man
3. A 25-year-old woman
4. A middle-aged adult
5. An elderly
6. No difference (the risk is comparable)

© 2021 Bastiani L et al. *JAMA Network Open.*

C. Communication

C1. How do you evaluate your knowledge about the risks associated with the use of ionizing radiation for medical purposes?

1. Excellent
2. Good
3. Fair
4. Sufficient
5. Inadequate

C2. From which communication channels have you usually received information about the risks associated with the use of ionizing radiation for medical purposes?
(multiple answers allowed)

1. TV/radio
2. Magazine/Newspaper
3. Internet or social media (Facebook, etc.)
4. Booklets
5. School, University
6. I have never received any information about ionizing radiation

C3. If you underwent a diagnostic examination with ionizing radiation, did you receive information about the risks associated with the use of ionizing radiation for that examination?

1. Yes
2. I have never received any information about ionizing radiation

C4. From which of the following would you like to receive information regarding the risks associated with the use of ionizing radiation for medical purposes? (multiple answers allowed)

1. TV/radio
2. Magazine/Newspaper
3. Internet or social media (Facebook, etc.)
4. Booklets
5. School, University

© 2021 Bastiani L et al. *JAMA Network Open.*

6. Healthcare professionals

C5. In the healthcare environment, from which professional would you prefer to receive information about the risks associated with the use of ionizing radiation? (multiple answers allowed)

1. Radiologist
2. Medical physicist
3. Radiographer
4. General practitioner

C6. At the end of a radiological exam, how would you prefer to be informed about the amount of radiation received? In terms of: (multiple answers allowed)

1. The radiation value expressed in terms of radiation units (i.e. 10 milliSieverts)
2. The equivalent risk to a given number of smoked cigarettes
3. The equivalent risk to a given number of days of background radiation exposure
4. The equivalent risk to a given number of kilometers traveled by car
5. I don't want to be informed

© 2021 Bastiani L et al. *JAMA Network Open.*

eTable 1. Literature References Used for Generation of the Survey Items

Year	First author	Country	Title	Patients (N=)	Items (N=)
2018	Al Ewaidat	Jordan	Knowledge and awareness of CT radiation dose and risk among patients	600	24
2015	Alhasan	Jordan	Medical radiation knowledge among patients in local hospitals	400	10
2017	Al-Mallah	Bahrain	Awareness and knowledge of ionizing radiation risks between prescribed and self-presenting patients for common diagnostic radiological procedures in Bahrain	416	20
2011	Baumann	United States	Patient perceptions of computed tomographic imaging and their understanding of radiation risk and exposure	1168	15
2016	Bohl	United States	Patient knowledge regarding radiation exposure from spinal imaging	99	14
2016	Gemechis	Ethiopia	Knowledge about radiation related health hazards and protective measures among patients waiting for radiologic imaging in Jimma University Hospital, Southwest Ethiopia	388	21

© 2021 Bastiani L et al. *JAMA Network Open.*

2013	Hartwig	United States	Parental knowledge of radiation exposure in medical imaging used in the pediatric emergency department	342	not reported
2019	Kenny	Ireland	Perception of medical radiation risk in Ireland: results of a public survey	326	14
2019	Lambertova	Czech Republic	Patient awareness, perception and attitude to contrast-enhanced CT examination: implications for communication and compliance with patients' preferences	263	17
2017	Lumbreras	Spain	Avoiding fears and promoting shared decision-making: how should physicians inform patients about radiation exposure from imaging tests?	602	14
2019	Oikarinen	Finland	Parents' received and expected information about their child's radiation exposure during radiographic examinations	41	13
2016	Repplinger	United States	Emergency department patients' perceptions of radiation from medical imaging	500	12
2017	Ria	Italy	Awareness of medical radiation exposure among patients: a patient survey as a first step for effective communication of ionizing radiation risks	737	13
2013	Ricketts	United States	Perception of radiation exposure and risk among patients, medical students, and referring physicians at a tertiary care community hospital	127	not reported

© 2021 Bastiani L et al. *JAMA Network Open.*

2018	Salerno	Italy	Complete written/oral information about dose exposure in CT: is it really useful to guarantee the patients' awareness about radiation risks?	430	39
2017	Schuster	United States	Awareness of radiation risks from CT scans among patients and providers and obstacles for informed decision-making	101	18
2012	Sin	Hong Kong	Assessing local patients' knowledge and awareness of radiation dose and risks associated with medical imaging: a questionnaire study	173	28
2016	Singh	Australia	A snapshot of patients' awareness of radiation dose and risks associated with medical imaging examinations at an Australian radiology clinic	238	14
2010	Takakuwa	United States	Knowledge and attitudes of emergency department patients regarding radiation risk of CT: effects of age, sex, race, education, insurance, body mass index, pain, and seriousness of illness	383	6
2014	Youssef	United States	Emergency department patient knowledge, opinions and risks tolerance regarding computed tomography scan radiation	409	25
2014	Zwank	United States	Emergency department patient knowledge and physician communication regarding CT scans	200	not reported

© 2021 Bastiani L et al. *JAMA Network Open.*

eTable 2. Psychometric Properties of the 10-Item KIRQ

Standardized factor loadings, internal consistency and goodness-of-fit indexes of the 10-item KIRQ						
	Coeff.	Std. Err.	z	P>z	[95% CI]	
B4. Natural sources of ionizing radiation	0.342	0.029	11.62	0.000	0.284	0.400
B5.a Ultrasound	0.202	0.026	7.27	0.000	0.140	0.244
B5.b Computed tomography	0.478	0.027	17.61	0.000	0.425	0.532
B5.c Magnetic resonance	0.382	0.025	15.49	0.000	0.333	0.430
B5.d Mammography	0.284	0.026	10.72	0.000	0.336	0.232
B6. Amount of radiation used	0.374	0.025	14.97	0.000	0.325	0.423
B7. Radiation emission after the examination	0.313	0.026	11.87	0.000	0.261	0.364
B8. Patient body size and amount of radiation	0.204	0.026	7.71	0.000	0.152	0.256
B9. Perception of potential danger of radiological tests using ionizing radiation	0.301	0.025	11.21	0.000	0.253	0.350
B10. Subjects at risk for ionizing radiation	0.282	0.026	10.930	0.000	0.231	0.332
Internal consistency: Cronbach α = 0.742 (CI95 0.706–0.796)						
Goodness of fit indexes: Standardized Root Mean Square = 0.018, Root Mean Square error of Approximation = 0.026, Comparative Fit Index = 0.958, Tucker-Lewis index = 0.927						

© 2021 Bastiani L et al. *JAMA Network Open.*
